# Factors influencing pharmacists and pharmaceutical scientists’ membership in professional organisations: an international survey

**DOI:** 10.1186/s40545-023-00620-6

**Published:** 2023-09-25

**Authors:** Louisa Sullivan, Jun Ni Ho, You Zhuan Lee, Garba Mohammed Khalid, Anisha Kaur Sandhu, Sherly Meilianti, Farah Aqqad, Renly Lim

**Affiliations:** 1 International Pharmaceutical Federation, The Hague, The Netherlands; 2 Oregon Health and Sciences University, Portland, OR USA; 3 Quality Use of Medicines and Pharmacy Research Centre, UniSA Clinical and Health Sciences, University of South Australia, Adelaide, 5000 Australia; 4 School of Pharmaceutical Sciences, Universiti Sains Malaysia, Gelugor, Penang Malaysia; 5 Pharmaceutical Engineering Group, School of Pharmacy, Queen’s University Belfast, Belfast, UK; 6 Monash University Malaysia, Jalan Lagoon Selatan, 47500 Bandar Sunway, Selangor Darul Ehsan Malaysia; 7 School of Pharmacy, University College London, London, UK

**Keywords:** Pharmaceutical societies, Health personnel, Needs assessment, Pharmacists, Professional organisations

## Abstract

**Background:**

Professional organisations exist as international or national organisations, with each country establishing at least one national professional association. There remains a knowledge gap about factors that influence professional organisational involvement of pharmacists and pharmaceutical scientists. This study aims to explore the motivators and barriers of pharmacy professionals holding organisation membership from a global perspective.

**Methods:**

An online questionnaire was developed and disseminated between May and July 2021. The survey was open to all pharmacists and pharmaceutical scientists. The survey consisted of four sections; demographic information, questions about professional organisations, about the International Pharmaceutical Federation (FIP) and its impact on the members. Data were analysed descriptively.

**Results:**

A total of 1033 complete survey responses were received and included in the analysis. Of all respondents, 761 (73.7%) respondents were current members of a professional organisation and 272 (26.3%) were not members of any professional organisation. Overall, findings demonstrated networking, education, training and professional development opportunities as the main interests and anticipated activities, while the lack of clarity or need to join organisation, time, and financial constraints as the main barriers of pharmacy professionals holding membership. The majority of FIP members are satisfied with current FIP activities, and anticipate further networking opportunities, educational resources and grants made available to members.

**Conclusions:**

Understanding the perceptions and needs, as well as factors that influence engagement of pharmacists and pharmaceutical scientists is the key to enhancing membership. Professional organisations are highly encouraged to strengthen and target activities according to the identified motivators and barriers.

**Supplementary Information:**

The online version contains supplementary material available at 10.1186/s40545-023-00620-6.

## Background

A professional organisation, often referred to as a professional body or professional association, is a collection of individuals within the same or related field of profession with a mission to support the interests of people working in that profession, to advance the profession, and to serve the public interest related to that profession. Professional organisations usually require membership fees, operate with an elected leadership body, and include a range of subcommittees or functional specialty areas. Professional organisations can be national or international, and often have close ties to other governmental and non-governmental organisations and/or agencies, including academic institutions that offer degrees or programs in that field of speciality [1, 2].

Within the profession of pharmacy and pharmaceutical sciences, the International Pharmaceutical Federation (FIP) is a global professional association which represents four million members of the profession. FIP has been registered as a non-governmental organisation with the World Health Organization since 1948, and aims to advance pharmacy profession through innovation in science, pharmacy practice, pharmacy workforce and education [3]. FIP also has a subgroup under its operation—the Early Career Pharmaceutical Group (ECPG) that actively supports early career pharmacists and pharmaceutical scientists in professional innovation and career development.

A professional organisation offers numerous benefits, including grant opportunities, accessibility to valuable resources and healthcare legislative information, and professional development, targeting members at different career stages, in particular early career [4–6]. Looking through the lens of pharmacy professional organisation, Fusco et al. [7] identified factors that motivated pharmacy students in organisation participation. The findings were consistent with those reported by Petersen et al. [5] as they characterised students’ area of interest in the organisation, networking opportunities, provision of student programmes and career development tools, as well as scholarships as important factors influencing pharmacy students’ decision in organisation involvement. Recent literature demonstrated similar findings, as continuing education, advancement in pharmacy practice opportunities, email updates and networking opportunities were indicated as significant influential factors [8]. While evidence highlight the importance and benefits of joining professional organisations, there appears to be a stagnant growth in membership for voluntary professional associations [9]. The perceived barriers such as generational issues, time and cost constraints may impact professionals’ decisions to hold membership [5, 9]. Hence, further understanding of the evolving needs and preferences of pharmacy professionals is imperative.

A critical gap remains as current knowledge about professional organisation membership involving pharmacists and pharmaceutical scientists from an international perspective is unknown. Existing studies either involved only pharmacy students at national level or were conducted at national level with limited study population. Roccograndi et al. [9], Liang et al. [10] included pharmacy graduates in their studies; however, those studies were deemed limited due to small sample sizes. There is a paucity of literature describing the elements that influence participation of pharmacists and pharmaceutical scientists in professional organisations, particularly the FIP. This study with a larger sample size representing pharmacists and pharmaceutical scientists of diverse background is critical to explore their perception of professional organisation involvement. The aims of this study are threefold: (i) to investigate reasons why pharmacists and pharmaceutical scientists join or not join professional organisation, (ii) to understand how they decide to join their preferred organisation, and (iii) to explore the views of FIP members about the organisation.

## Methods

### Setting

This cross-sectional study was conducted via an online survey from 24 May 2021 to 5 July 2021 (6 weeks) by a FIP ECPG working group consisting of five early career pharmacists and pharmaceutical scientists. Notably, ECPG was known as FIP Young Pharmacists’ Group (YPG) during the survey period.

### Participants

The survey was open to all pharmacists and pharmaceutical scientists.

### Instrument development and dissemination

The questionnaire was developed by the project team and consisted of a combination of open- and close-ended questions. The first draft of the questionnaire was reviewed by the FIP staff and was refined prior to piloting with the FIP YPG Subcommittee 2021 (*n* = 20). The pilot phase led to minor modifications in the questionnaire, primarily on the order of the questions and clarity of the language. The final survey consisted of four sections; demographic information (six questions), questions about professional organisations (with sub-questions depending on whether the respondents were part of a professional organisation), about FIP and YPG membership (with sub-questions depending on whether the respondents were part of FIP and YPG) and impact of FIP and FIP YPG on the members (Additional file 1, Fig. 1).

**Fig. 1:**
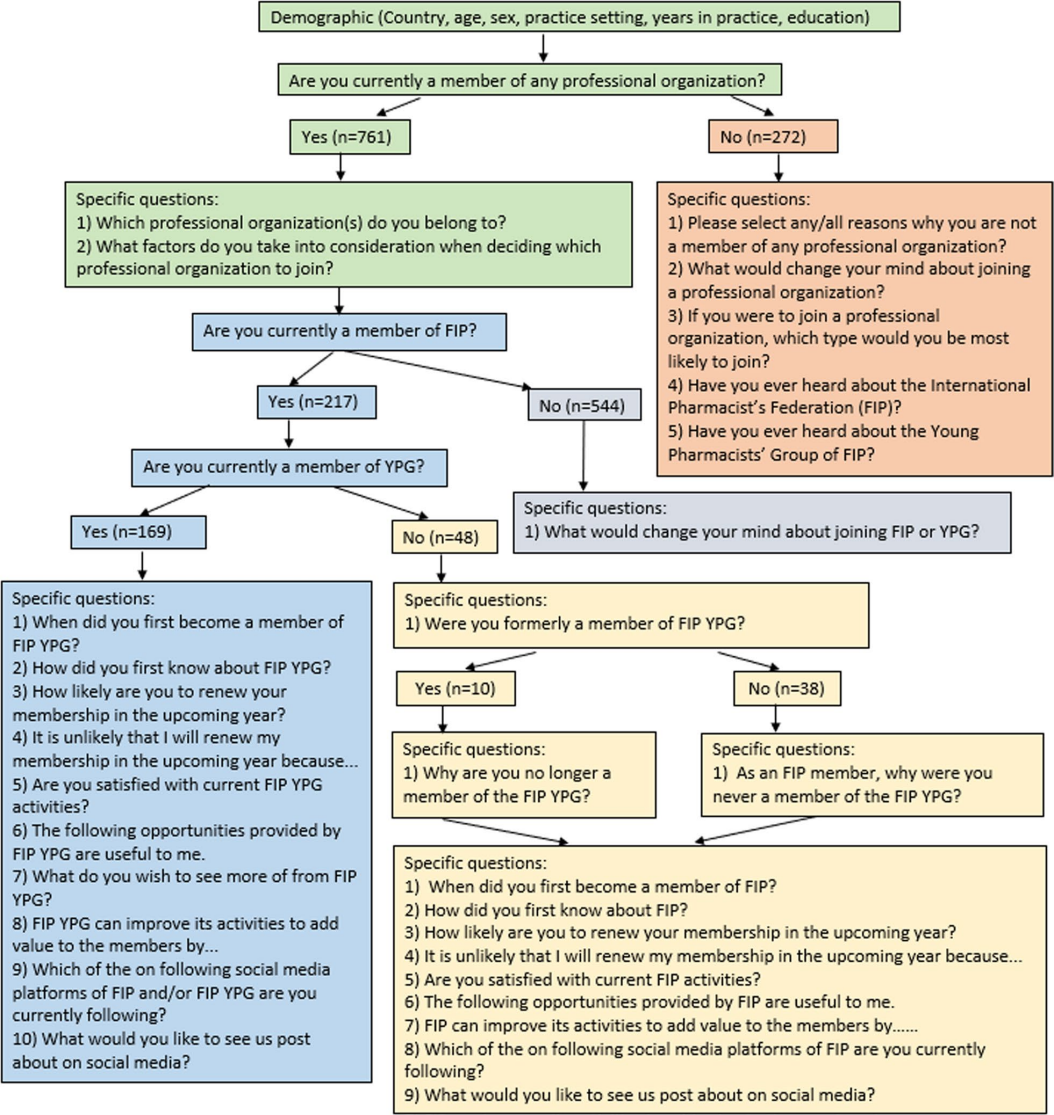
Flowchart of questionnaire responses

The questionnaire was developed as a web-based survey using the QuestionPro online survey platform. Participants were required to complete all responses and would be prompted to complete questions that were not answered before proceeding to the following page. Respondent’s IP address was used to identify a unique survey response. The survey was disseminated using social media platforms such a LinkedIn, Facebook, Instagram, and Twitter as well as via Mailchimp® (Atlanta, Georgia, USA) email service to all members of the FIP YPG.

### Data analyses

All data were exported from QuestionPro into Microsoft Excel. Data were analysed using Microsoft Excel (Additional file 2).

### Ethical consideration

This study was approved by the University of South Australia Human Research Ethics Committee (Application ID: 203866). Respondents were asked to fill in the survey voluntarily and were not asked to disclose their identity.

## Results

### Demographic characteristics

A total of 1033 complete survey responses were received and included in the analysis (Table 1), with 425 male respondents (41.1%), 576 female respondents (55.8%) and 32 (3%) unknown (not answered). The age range was between 18 and 70 years, with a median of 29 years. The majority were from the African (*n* = 249, 24.1%), Western Pacific (*n* = 240, 23.2%) and Southeast Asian regions (*n* = 214, 20.7%). Most respondents worked either in a hospital (*n* = 252, 24.4%), community setting (*n* = 218, 21.1%) or academia (*n* = 172, 16.7%). The survey covered respondents across the six WHO regions and is representative of pharmacists from all regions.

**Table 1:** Demographic characteristics of survey respondents

Demographics	Respondents
Gender (*n*, %)	
Male	425 (41.1%)
Female	576 (55.8%)
Unknown	32 (3%)
Age (median, range)	29 (18–70)
Years of practice (median, range)	3 (0–50)
WHO region	
Africa	249 (24.1%)
Americas	140 (13.6%)
Eastern Mediterranean	38 (3.7%)
Europe	152 (14.7%)
Southeast Asia	214 (20.7%)
Western Pacific	240 (23.2%)
Practice setting	
Academia	172 (16.7%)
Community	218 (21.1%)
Hospital	252 (24.4%)
Industry	99 (9.6%)
Regulatory	51 (4.9%)
Students	140 (13.6%)
Others	101 (9.8%)

Of the 1033 respondents, 761 (73.7%) respondents were current members of a professional organisation and 272 (26.3%) were not members of any professional organisation. Of the 761 respondents with a current professional organisation membership, 217 (28.5%) were current members of the FIP, including 169 current members of the FIP YPG. Of the remaining 48 FIP members, 10 were former members of the FIP YPG, while 38 were not (Fig. 1).

### Professional organisation membership

Of the 761 respondents who were current members of a professional organisation, 217 (28.5%) were members of FIP, 91 (11.8%) members of the International Pharmaceutical Students Federation, 77 (10.1%) members of regional organisations, 578 (75%) members of their national professional organisations and 249 (32.7%) members of their local professional organisations. Note: total number exceeds 761, because respondents can be members of more than one professional organisation.

### Reasons for joining a professional organisation (*n* = 761)

Of the 761 respondents who were members of professional organisations, about two-thirds (*n* = 503, 66%) of respondents named the mission of the organisation as their primary motivation for involvement. Other important factors that were taken into consideration included access to educational content and resources (*n* = 487, 63.9%), networking opportunities (*n* = 449, 58.9%) and continuing professional development (43.8%). Over a third (*n* = 280, 36.8%) of respondents identified membership fees as one of the deciding factors.

### Reasons for not joining a professional organisation (*n* = 272)

Of the 272 respondents who were not members of any professional organisations, the key reason for respondents not holding a membership was their lack of clarity in need or purpose of joining a professional organisation (*n* = 93, 34.1%). Other factors were financial concerns (*n* = 62, 22.7%), lack of awareness of organisations’ existence (*n* = 54, 19.9%), and time constraints (*n* = 51, 18.8%), which contributed to respondents’ non-involvement in a professional organisation.

When asked about elements that would influence respondents’ re-evaluation of decisions in professional organisation involvement, respondents reported access to improved education as the primary motivator (*n* = 138, 50.7%). Other important motivation factors were more networking opportunities (*n* = 129, 47%), reduced membership fees (*n* = 101, 37.1%), increased availability of grants or scholarships (*n* = 90, 33.1%), and more opportunities to serve on committees (*n* = 88, 32.4%).

When asked which type of organisations (international, regional, national, or local) the respondents would consider joining, over half (*n* = 164, 60%) said they would join an international organisation if they were to join a professional organisation. About half (*n* = 137, 50%) of the respondents would join a national organisation, while about a third or less would join a regional (*n* = 87, 32%) or local (*n* = 68, 25%) organisation.

### Views of current FIP members about the FIP (*n* = 48)

Of the 48 FIP members who are not part of the YPG (yellow shading in Fig. 1), most respondents first learned about FIP through friends or colleagues (*n* = 15, 31.3%), through national or regional organisations (*n* = 15, 31.3%) and during study at university before graduation (*n* = 9, 18.8%). Most respondents reported very likely or likely to renew their membership in the upcoming year (*n* = 40/41, 97.6%).

In terms of level of satisfaction with the current FIP activities, the majority indicated being either very satisfied or satisfied (*n* = 38/40, 95%), with no responses recorded for unsatisfied and very unsatisfied. Most FIP members found networking opportunities provided by the FIP to be useful (*n* = 36/41, 87.8%). Overall, most respondents agreed or strongly agreed that professional networking with national and international members (*n* = 36/41, 87.8%) and collaborative research opportunities were useful (*n* = 31/41, 75.6%). Other reported useful activities were congresses and grants, resources and contributing and learning opportunities.

Survey respondents were also asked about what activities they would like to see more of from FIP. The most anticipated activity by the survey respondents was networking opportunities (*n* = 22, 45.8%). Other anticipated opportunities include availability of travel grants to congresses (*n* = 18, 37.5%), collaborative research (*n* = 17, 35.4%), access to educational content and resources (*n* = 15, 31.3%), as well as research grants and scholarships (*n* = 15, 31.3%).

The most popular social media platform that FIP members follow are LinkedIn (*n* = 20, 41.7%) and Twitter (*n* = 20, 41.7%), followed by Facebook (*n* = 19, 39.6%). When questioned about the type of content that FIP members would like to see more of, respondents indicated that they would prefer upcoming virtual and face-to-face events being shared on social media (*n* = 27, 56.3%), followed by opportunities for grants or scholarships (*n* = 22, 45.8%), as well as health-related news (*n* = 18, 37.5%).

### Views of current YPG members about FIP YPG (*n* = 169)

From the YPG perspective, of the 169 responses received (blue shading in Fig. 1), 133 (78.7%) have joined the FIP YPG as members for the past 5 years. Social media played a vital role in promoting YPG, with 27.8% (*n* = 45, 26.6%) of respondents learning about YPG through this platform, while 23.7% (*n* = 40) of them through the FIP-based digital events, conferences, or promotional emails. About 16% (*n* = 27) were introduced to the FIP YPG through university pharmacy organisations or the International Pharmaceutical Students Federation (IPSF).

Most respondents indicated they were likely to very likely to renew their membership in the following year (*n* = 156/168, 92.9%). A small number of respondents (*n* = 7/168, 4.2%), however, stated that they would prefer not to renew their membership in the coming year, citing expensive fees, lack of clear benefit or interest as reasons.

Respondents were asked if they were satisfied with current FIP YPG activities, where 76.6% (*n* = 115/150) were either satisfied or very satisfied. More than 90% of members (*n* = 154) responded that YPG opportunities, such as networking; professional and personal development; digital events and conferences; continuing education and mentorship; grant awards; research, volunteering or committee-serving opportunities were useful to them. FIP YPG members were most interested in more professional development (*n* = 106, 62.7%), networking (*n* = 105, 62.1%) and travel or research grants and scholarship opportunities (*n* = 90, 53.3%).

Amongst YPG members, the top three social media platforms found to be most popular for following FIP YPG-related news were Facebook (62.7%, *n* = 106), LinkedIn (*n* = 88, 52.1%) and Instagram (*n* = 88, 52.1%). This was followed closely by Twitter (*n* = 62, 36.7%), while YouTube was seldom used as a source for FIP YPG information (*n* = 20, 11.8%). FIP YPG members would like to receive more information on virtual or face-to-face events (*n* = 124, 73.4%), grant, scholarship, volunteering, or mentoring opportunities (*n* = 138, 81.7%); and health-related news (*n* = 105, 62.1%) on the FIP YPG social media platforms.

## Discussion

This study is the first publication to survey and report views of pharmacists and pharmaceutical scientists from an international perspective about their involvement in a professional organisation. Our findings demonstrate that while most respondents hold at least one current membership, there is still a small proportion who do not.

Our results suggest that most respondents value the mission of organisation, easy accessibility to educational resources, networking, and professional development opportunities, all of which are important factors that lead them to hold current professional organisation membership. The largest proportion of respondents valued mission of organisation, reporting it as one of the important motivators. Membership is likely to expand when an organisation defines clear objectives and demonstrates its contributions to members, as previous studies has shown that there is a positive correlation between organisational values and employee engagement [11–14]. A potential enabler to improving organisational performance and boosting members’ participation would be cultivating an effective organisational culture that aligns with its mission.

The factors influencing membership in professional organisations that our study found were similar to factors found in other studies; however, only a few had organisation mission as one of key considerations to participating in professional organisation [8]. Area of interest and networking opportunities were the most important factors influencing the decision to join a professional organisation among pharmacy students [5, 7], while continuing education and opportunity for pharmacy practice advancement were the main reasons for pharmacy graduates to be involved in professional organisations [8]. Similarly, the most significant factors that encourage membership in professional organisation within the nursing profession were continuing education and improvement of the profession [4, 15].

As the pharmacy profession undergoes a rapid transformation from traditional dispensing to patient-centred care delivery, pharmacists must now maintain their competencies by keeping abreast of medicines-related knowledge, including but not limited to pharmacy practice, pharmaceutical sciences and professional standards legislation and regulations [16]. This potentially contributes to one of the reasons pharmacy professionals join an organisation to access educational resources. Our results reflect the needs for networking, leadership opportunities, and professional and career development, all of which are consistent with a prior study investigating the opinions and expectations of the FIP YPG members [17]. With these needs being proposed by current and prospective members, we also suggest putting greater emphasis on acquiring and developing transferrable skills for personal and career growth.

The lack of resources such as time and costs are the top reasons hindering students, graduates, and professionals within and across different profession from joining professional organisations [5, 7, 15, 18, 19]. While time and financial constraints are commonly known reasons for not holding professional organisation membership [5], the lack of clarity or need of joining and awareness of organisation’s existence is unexpected in our study. Such information is somehow contradictory to the highly valued outlined mission of organisation by current members, as previously discussed. Similar to our findings, a cross-sectional study identified a lack of perceived benefit from the organisation as one of the main factors of pharmacy students discontinuing membership, while a qualitative study involving chiropractors highlighted the lack of direct benefit and clear reasons for being a member as deterrent factors [7, 19]. Organisations should take these findings into evaluation in refining their goals and developing strategies to engage new members. They may consider introducing a change or adjustment to the membership fees, promoting more grant and leadership opportunities, and providing more education and training support as these have been identified as the preferred activities and interests which may lead to respondents reconsidering participation in a professional organisation. Access to a suite of education and training support has been shown to positively improve job and career satisfaction, which may help sustain an effective workforce and secure pharmacy practice future [20].

Most respondents reported getting to know about FIP through their friends or colleagues, or from their regional or national professional bodies. Others became aware of the FIP activities from their undergraduate programs organised by the IPSF, and some through social media platforms and promotional emails. The increase in FIP membership in the last decade could be explained by the expanded presence and impact of FIP at regional levels since 2019 due to the FIP regional conferences. The regional conferences aimed to extend the support of FIP through regional programs and promote the role of pharmacists in the health care system. For instance, the first two regional conferences were held in mid and late 2019 in Amman, Jordan, and Ankara, Turkey for Eastern Mediterranean and the European region, respectively. Since the COVID-19 pandemic, digital events organised by FIP have increased more than 100-fold, and promotion of these events via different social media platforms has also proportionately increased, thus increasing the visibility of FIP and the awareness of its activities worldwide. Several studies have suggested the role of social media communication towards boosting the identity of professional organisations, promotion of open innovation in digital health, and supporting work engagement, and social support [21–23]. Our findings align with these studies as both the FIP and YPG members actively employ social media as the key platforms to keep themselves updated with contemporary knowledge.

Retaining membership is an annual commitment by the FIP members, and from our findings, an appreciable number of the FIP and YPG members have expressed their interest in renewing the annual membership. Given the overall positive responses, most of them are either satisfied or very satisfied with the current FIP activities, particularly those providing national and international networking opportunities, and collaborative research opportunities. These highly valued opportunities include the FIP world annual congress and regional conferences, access to educational content and e-resources, digital events, online courses, volunteerism opportunities, mentorship programmes, as well as personal and professional development initiatives. Despite their satisfaction with the current activities, both YPG and FIP members still anticipate further networking and engagement opportunities. The YPG members’ interests lie in education and training opportunities, while the FIP members’ interests involve increasing the reputation of pharmacy profession. Professional priorities vary across the career span, thus, it is unsurprising early career pharmacy professionals entering the workforce anticipate additional educational training and financial support, while middle-to-late-career pharmacy professionals tend to engage in advancing the profession.

There are limitations to this study. First, the survey was conducted only in English. There is a possibility that the questions may have been misinterpreted by respondents from countries, where English is not their first language. Second, although we received over a thousand survey responses, the large number of pharmacists and pharmaceutical scientists globally means that our survey captured only a very small percentage of these professionals. Third, an association between variables such as qualification, gender and time involved in activities and organisational involvement was not explored. Previous studies suggested potential difference between these variables and membership, and hence additional research should be conducted to determine whether membership varies with education level, gender, and the time for commitment to organisational activities. Finally, the survey was mainly targeted towards early career pharmacists and pharmaceutical scientists, and therefore, the findings may not be generalisable to the whole profession.

## Conclusions

Considerations around holding professional organisation membership involve many factors and are specific to the interests and needs of pharmacists and pharmaceutical scientists. This global study provides guidance about pharmacy professionals’ views and expectations of joining professional organisations. Understanding their perceptions and values, as well as factors that influence their engagement helps professional organisations in enhancing membership through targeting activities according to the identified motivators and barriers. Organisations should employ strategies tailored to members’ needs to better align their goals. Ultimately, valuable information from this study help promote and achieve the goals of FIP to transform and advance the pharmacy profession over the next decade globally, regionally, and nationally.

### Supplementary Information

**Additional file 1.** Questions included in the survey.

**Additional file 2.** Checklist for Reporting of Survey Studies (CROSS).

## Data Availability

All data generated or analysed during this study are included in this published article and its supplementary information files.
