# Supplementary material for: Factors influencing pharmacists and pharmaceutical scientists’ membership in professional organisations: an international survey
Source: J Pharm Policy Pract. 2023 Sep 25;16:105. doi: 10.1186/s40545-023-00620-6 (PMC10521542; doi:10.1186/s40545-023-00620-6)
Supplement: Supplementary file 1 — Additional file 1. Questions included in the survey. [file 40545_2023_620_MOESM1_ESM.pdf]

Section A, demographic information

Q1 Country of residence

[Select from dropdown]

(see page 7-11 for list of countries)

Q2 Age

a. 20-24
b. 25-29
c. 30-34
d. 35-39
e. 40-44
f. 45-49
g. 50 and above

Q3 Gender

a. Male
b. Female
c. Prefer not to say

Q4 Current practice setting

a. Academia
b. Hospital
c. Community
d. Industry
e. Regulatory
f. Military or paramilitary
g. Others (specify)

Q5 Number of years in practice

[dropdown options in years]

Q6 Highest level of education

a. PharmD
b. B.Pharm
c. MPharm
d. Master's
e. Ph.D.
f. Others (specify)

Section B, about professional organization

Q1 Are you currently a member of any professional organization?

a. Yes
b. No

Q1A What factors do you take into consideration when deciding which professional organization to join? (select all that apply)

A. Educational content and resources
B. Networking opportunities
C. Volunteering opportunities
D. Opportunities for grants and/or scholarships
E. Opportunities to serve on committees
F. Content and quality of conference
E. Annual membership fee
F. Current board committee members
G. Others (please specify)

Q1B Which professional organization(s) do you belong to? (select all that apply)

a. International Pharmaceutical Federation (FIP)
b. International Pharmaceutical Students' Federation (IPSF)
c. Regional (multiple countries included) pharmacy organization
d. National pharmacy organization in my country
e. Local pharmacy organization (in my city or state)
f. Others (please specify)

Q1C Please select any/all reasons why you are not a member of any professional organization?

a. Unaware of any professional organization
b. Not clear about the purpose or the need to join a professional organization
c. Uninterested
d. Not seeking additional support or networking
e. Financial constraints
f. Do not have the time
g. Other (please specify)

Q1D What would change your mind about joining a professional organization? (select all that apply)

a. Better educational content and resources
b. More networking opportunities
c. More volunteering opportunities
d. More grants and/or scholarships
e. More opportunities to serve on committees
f. Better content and quality of conference
g. Reduced Membership fee
h. None
i. Others (please specify)

Q1E If you were to join a professional organization, which type would you be most likely to join?

a. International/global
b. Regional
c. National
d. Local

Q2 Have you ever heard about the International Pharmacist's Federation (FIP)?

a. Yes, I have heard the name, but I don't know much about it.
b. Yes, I am aware of the organization, but I am not interested in joining

c. No, I haven't heard of it

Q2A Have you ever heard about the Young Pharmacists' Group of FIP?
a. Yes, I have heard the name, but I don't know much about it
b. Yes, I am aware of the organization, but I am not interested in joining
c. Yes, I would like to join, but I am not eligible
d. No, I haven't heard of it

Q2B What would change your mind about joining FIP or YPG? (select all that apply)
a. Better educational content and resources
b. More networking opportunities
c. More volunteering opportunities
d. More grants and/or scholarships
e. More opportunities to serve on committees
f. Better content and quality of conference
g. Reduced membership fee
h. None
i. Others (please specify)

Section C, about FIP and YPG membership

Q1 Are you currently a member of FIP YPG?
[Yes] [No]

Q1A When did you first become a member of FIP YPG (please select approximate year)?
[dropdown options in years, e.g 2000, 2001, 2002 to 2021]

Q1B For how long have you been a member of FIP YPG (please indicate approximate years you have an active membership)?
[dropdown options in years from 1 to 15]

Q1C How did you first know about FIP YPG?
a. Through social media (Facebook, Twitter, Instagram, etc.)
b. Through promotional emails
c. Through a friend
d. From my university
e. From my workplace
f. Through a national or regional YPG
g. Others (please specify)

Q1D How likely are you to renew your membership in the upcoming year?
a. Very likely
b. Likely
c. Unlikely

Q2 Are you formerly a member of FIP YPG?
[Yes] [No]

Q2A Why are you no longer a member of the FIP YPG?
a. No longer meet the eligibility criteria
b. I do not see the benefit of being a YPG member
c. Others (please specify)

Q2B As an FIP member, why were you never a member of the FIP YPG?
a. Did not meet criteria to join FIP YPG at time of first FIP membership
b. FIP YPG was not founded until after I was no longer eligible for membership
c. I did not see the benefit of being a YPG member

d. Other (please specify)

Section D, the impact of FIP and FIP YPG on the members

Q1 Are you satisfied with current FIP YPG activities?

a. Very satisfied
b. Satisfied
c. Neutral
d. Unsatisfied
e. Very unsatisfied

Q2 The following opportunities provided by FIP YPG are useful to me.

	Strong disagree	Disagree	Neither Agree nor Disagree	Agree	Strongly agree	Unsure
Educational content and resources						
International contacts						
Networking opportunities						
Volunteering/ externship opportunities						
Research grants and/or scholarships						
Travel grant						
Mentorship programme						
Online workshops and/or digital events						
Opportunities to serve on committees						
Personal development						
Professional development						

Q3 What do you wish to see more of from FIP YPG? (multiple choice)

a. Educational content and resources
b. Networking opportunities
c. Volunteering/externship opportunities
d. Research grants and/or scholarships
e. Mentorship opportunities
f. Online workshops and/or digital events
g. Opportunities to serve on committees
h. Other (please, specify)

Q4 How best do you think FIP YPG activities could be improved to maximize impacts on its members? (open-ended)

Brief answers only

Q6 Which of the on following social media platforms of FIP and/or FIP YPG are you currently following?

a. Facebook
b. Twitter
c. LinkedIn
d. Instagram
e. Youtube
f. None

Q7 What would you like to see us post about on social media?

a. Upcoming virtual events
b. Opportunities on grants, scholarships, volunteering, mentorship etc
c. Health-related news
d. Others (please specify)

THANK YOU FOR YOUR TIME!
